# Disparities in perceptions of long-acting reversible contraceptives among patients in Delaware Title X clinics^☆^

**DOI:** 10.1016/j.contraception.2025.111002

**Published:** 2025-06-26

**Authors:** Collin W. Mueller, Constanza Hurtado-Acuna, Heide M. Jackson

**Affiliations:** a Department of Sociology, University of Maryland-College Park, College Park, MD, USA; b Carolina Population Center, University of North Carolina-Chapel Hill, Chapel Hill, NC, USA; c Maryland Population Research Center, University of Maryland-College Park, College Park, MD, USA

**Keywords:** Contraceptive attitudes, LARC, Patient-provider trust, Racial/ethnic disparities, Title X

## Abstract

**Objectives:**

We examined whether there were racial/ethnic and insurance status differences in patients reporting a sense of relief with the idea of using long-acting reversible contraceptives (LARCs) and whether these differences were attenuated by provider trust and fertility concerns among Title X patients.

**Study design:**

We analyzed two waves of data from a repeated cross-sectional patient survey designed to capture a representative sample of Title X patient health encounters in Delaware (N = 797), fielded in the context of a larger study designed to evaluate a statewide intervention to improve patient access to highly effective contraceptive methods.

**Results:**

We found that a majority of patients reported a sense of relief associated with the idea of using LARCs and differences by race/ethnicity and insurance status. Insurance coverage differences were attenuated by adjusting for reversibility concerns, fertility concerns, and patient-provider trust, but racial/ethnic disparities remained. Fifty-nine percent (95% CI, 55%–63%) of Black respondents reported a sense of relief at the idea of having an LARC, compared with 67% (95% CI, 63%–71%) and 65% (95% CI, 62%–67%) of White and Hispanic/Latinx patients, respectively. Patients who did not trust their provider to remove an IUD on request had a predicted probability of 50% (95% CI, 43%–57%) of viewing LARC with a sense of relief compared with 70% (95% CI, 64%–76%) of patients who reported trust in their provider to do so.

**Conclusions:**

This study contributes to the growing body of research on reproductive autonomy by shedding further light on how decisions regarding contraceptive usage are made in the context of perceived agency constraints in health care settings.

**Implications:**

This study underscores the importance of efforts to improve equitable access to long-acting reversible contraceptives by promoting patient-provider trust, addressing patient concerns regarding the short- and long-term effects of LARCs, and ensuring patients have continued access to healthcare treatment.

## 1. Introduction

Intrauterine devices (IUDs) and contraceptive implants are highly effective long-acting reversible contraceptive (LARC) methods and are used by a growing number of individuals; among women aged 15 to 44 years, usage increased from 6% to 17% over the last decade [1,2]. Unlike other methods extensively used (e.g., oral contraceptives and condoms), patients are highly dependent on providers for LARC insertion and removals. Limited patient and provider knowledge, lack of clinician training in insertion, and costs to patients present barriers to LARC use [3–5]. The Affordable Care Act’s Contraceptive Coverage Requirements that eliminated out-of-pocket costs for contraception (including LARC insertion and removals), state-based reimbursement policies, and clinician training have removed some barriers to LARC use [6–11]. Additionally, ambivalence about having a child, uncertainty about access to removals, and concerns about side effects deter individuals from using LARCs [4,12–14]. At the same time, providers sometimes promote LARC methods among those who do not desire to use contraception or who may prefer another contraceptive method [15] using criteria such as “risk of unintended pregnancy” or “poverty avoidance” as reasons to suggest LARCs to racial minorities and low-socioeconomic status patients [16–18]. Provider-level biases coupled with cost barriers to LARC removal may constitute forms of contraceptive coercion (i.e., behaviors that interfere with decision-making related to reproductive health), particularly for low-income and minoritized patients who have experienced a long history of contraceptive coercion in the United States [14,16,19–21].

Past studies about patients’ LARC attitudes and usage preferences are based on small samples of, in general, more educated patients, LARC users, and postpartum patients [22,23]. Studies have also focused more on IUDs than implants, which are equally effective and have less invasive insertion procedures [24]. Recent research has examined how people perceive IUDs vs implants and underscores the need to further clarify the relationship between racial/ethnic and socioeconomic status disparities in patient perceptions regarding types of LARCs and provider behaviors that undermine patient autonomy [25]. The present study advances this area of research using a probabilistic clinic-based sample of Black, Latinx, and White Title X patients from Delaware. We examine racial/ethnic disparities in patients reporting a sense of relief associated with the idea of using LARCs in relation to attitudes concerning condoms, IUD- and implant-specific reversibility and fertility concerns, and trust in providers to remove an IUD or implant when requested.

## 2. Data and methods

### 2.1. Context

The Delaware Contraceptive Access Now (DelCAN) initiative was launched in 2015 by Upstream USA and the state of Delaware to ensure same-day access to all contraceptive methods and long-acting reversible contraceptives (LARCs) in particular, regardless of a patient’s ability to pay. Upstream USA is a national nonprofit organization that partners with clinics to promote equitable access to patient-centered contraceptive care. From 2015 to 2019, the Delaware Division of Public Health funded the acquisition of LARC devices, which enabled clinics receiving funding through the Title X program (Title X clinics) to provide options for IUD and implant devices at no cost to individuals of any income level, therefore eliminating the sliding scale co-insurance fee that was previously in place for those above the poverty line. Additionally, the public awareness online and social media advertising campaign “Be Your Own Baby” was fielded from May 2017 to October 2018 to help patients find health care providers who could provide free or low-cost same-day contraceptives [26].

The main population that stood to gain access from reductions in contraceptive costs through DelCAN were uninsured patients seeking to access contraception who fell above 100% of the poverty line—a population primarily served by Title X providers. While DelCAN also covered LARC removal fees, low-income patients’ concerns regarding their continued access to care may represent a barrier to LARC usage. We were particularly interested in examining perceptions regarding LARCs among the Title X patient population, given the history of contraceptive coercion among minoritized patients and that the costs associated with LARC removal may continue to constitute a form of contraceptive coercion for low-income patients without adequate access to care [20,27].

### 2.2. Survey

The DelCAN evaluation conducted a repeated cross-sectional survey of patients who identified as female between the ages of 15 to 44 years and were seeking health care for themselves in a sample of Title X clinics in 2018 (wave 1) and 2019 (wave 2). Seven large Title X clinics were included in the sample, and four smaller clinics were randomly selected among 13 smaller Title X clinics operating in the state. Potential participants were recruited by researchers in clinic waiting rooms. After obtaining written consent, survey participants were asked about a variety of social, behavioral, and demographic factors, including their prior health care usage, trust in health care providers, perceived quality of health care communication, and knowledge and perceptions of the most effective contraceptive methods (i.e. implants and IUDs). Respondents provided self-reported answers via tablet before and after their health care encounters with providers. For our analysis, we used pre-encounter survey responses from both survey waves and restricted analysis to Black/African American, Hispanic/Latinx, and White individuals who consented to participate, were between the ages of 18 and 44 years, reported that they were never told by a health care provider that they were infertile, and reported answers to the question used to construct *LARC relief* (our key outcome variable described below; *N* = 797).

### 2.3. Key Variables

LARC relief, our dependent variable, was constructed by dichotomizing responses to the statement, “It’s a relief to have an IUD or implant that works without having to do anything.” It distinguishes between respondents who reported that they agree or strongly agree to the statement (1) from those who reported that they neither agree nor disagree, disagree, or strongly disagree (0). Race/ethnicity was constructed as a categorical variable corresponding with whether the respondent reported being non-Hispanic/Latinx Black (reference group), Hispanic/Latinx, or non-Hispanic/Latinx White. Uninsured status was constructed as a dichotomous variable corresponding with respondents who reported not being currently covered by any health insurance or health coverage plan (uninsured = 1, insured = 0). Educational status was constructed as a categorical variable with values corresponding with less than a high school education, high school graduate, some college, Associate’s degree, Bachelor’s degree, or more. We measured health care access and usage history using respondents’ self-reports regarding whether they had deferred health care in the last 12 months prior to the visit where they were interviewed and whether they had previously established care at the clinic where they were interviewed. We also coded a series of covariates measuring perceptions of other contraceptive methods and knowledge about LARCs. We constructed a dichotomous condom attitude variable, *belief that condoms are too much of a hassle*, distinguishing between those who reported that they agree or strongly agree (1) from those who reported that they neither agree nor disagree, disagree, or strongly disagree (0) with the statement, “It is too much of a hassle to use a condom every time I have sex.” IUD reversibility concerns were measured using True (=1) and False (=0) responses to the statement, “The IUD cannot be removed early if a woman changes her mind about wanting to get pregnant.” LARC fertility concerns were measured using True (=1) and False (=0) responses to the statements, “If you use an IUD it might affect your ability to have a child in the future, even if you have your IUD taken out,” and, “If you use an implant it might affect your ability to have a child in the future, even if you have your implant taken out.” We constructed dichotomous LARC-specific provider trust variables, *trust in provider to remove IUD/implant when requested* (trust=1; distrust=0) from responses to the statements, “My provider will remove my IUD whenever I decide I’d like it removed,” and, “My provider will remove my implant whenever I decide I’d like it removed.” These variables distinguish between those who reported that they agree or strongly agree to the statement (1) from those who reported that they neither agree nor disagree, disagree, or strongly disagree (0).

### 2.4. Analytic strategy

We used a series of logistic regression models to predict the likelihood that respondents reported LARC relief. Model 1 adjusts for race/ethnicity and age. Model 2 adds educational attainment (high school graduate, some college, associate’s degree, bachelor’s degree or higher) and insurance status (uninsured or insured), and health care access and usage history. Model 3 includes an adjustment for contraceptive attitudes (belief that condoms are too much of a hassle, belief that IUDs are not reversible, belief that IUDs cause infertility, beliefs that implants are not reversible, and beliefs that implants cause infertility). Model 4 adjusts for provider trust variables because provider trust may be associated with attitudes toward LARCs. All models also control for survey wave and use robust SEs to account for potential heteroskedasticity clustered by clinic location. We also ran race/ethnicity-stratified models to examine whether unique patterns emerged for racial/ethnic subsamples (i.e., examining whether racial/ethnic social position may operate as an effect modifier in the relationships between the other included variables and LARC relief); results were substantively similar, indicating that the relationships between variables of interest and LARC relief were similar across groups. We used multiple imputation by chained equations to impute missing item nonresponse data for all person-level independent variables and controls; we imputed 50 data sets and averaged the results of our regression analyses using Rubin’s rules with Stata/MP 17.0 [28]. Substantive results did not change regardless of whether we used multiple imputation or complete case analysis. For interested readers, we include Supplemental Tables noting the amount of data that is missing and percent imputed for each variable (Table S1) and providing Models 1 through 4 using only complete cases (Table S2). To facilitate interpretation of odds ratios, we calculate predicted probabilities of LARC relief from an unadjusted model and our fully adjusted Model (Model 4). When calculating the adjusted predicted probabilities, values for a group depend on the odds ratio and the values of control variables included in the model. We set all control variables to have the sample mean values following standard practice implemented with mimrgns command in Stata 17.0 (StataCorp LLC, College Station, TX).

## 3. Results

Table 1 provides descriptive statistics using the imputed data sets. Sixty-three percent of respondents reported LARC relief. Twenty-six percent of respondents were uninsured. About three in 10 respondents reported concerns that IUDs (27%) could not be removed if desired. More than half of respondents reported concerns that using an IUD (56%) or implant (65%) might adversely affect their fertility in the future. Seven in 10 respondents reported that they trusted that their provider would remove an IUD (69%) or implant (70%) upon request.

Table 2 reports unadjusted proportions of the sample reporting LARC relief across each categorical variable and the correlation between LARC relief and age. Sixty-one percent (95% CI, 55%–66%) of Black or African American respondents, 66% (95% CI, 60%–72%) of White respondents, and 64% (95% CI, 58%–70%) of Hispanic/Latinx respondents reported relief at the idea of having an IUD or implant. Sixty-four percent (95% CI, 60%–68%) of insured respondents and 61% (95% CI, 54%–68%) of uninsured respondents reported relief at the idea of having an IUD or implant.

Table 3 reports predicted probabilities from unadjusted and fully adjusted (Model 4) models. Supplemental Table S3 reports odds ratios and 95% CIs from a series of logistic regressions for the full sample. Across models, Hispanic/Latinx and White respondents were more likely to perceive LARCs as a source of relief relative to Black respondents. In the fully adjusted model, 59% (95% CI, 55%–63%) of Black respondents reported a sense of relief at the idea of having an LARC, compared with 67% (95% CI, 63%–71%) and 65% (95% CI, 62%–67%) of White and Hispanic/Latinx patients, respectively. Being uninsured was negatively associated with LARC relief, and this difference appeared to be attenuated after adjusting for provider trust (Model 4). Trust in providers to remove IUDs and implants when requested was strongly positively associated with the likelihood of reporting LARC relief. In the final model (Model 4), patients who did not trust their provider to remove an IUD on request had a predicted probability of 50% (95% CI, 43%–57%) of viewing LARC with a sense of relief compared with 70% (95% CI, 64%–76%) of patients who reported trust in their provider to do so.

## 4. Discussion

This research adds to a growing literature describing people’s perceptions of long-acting reversible contraceptives in relation to contraceptive knowledge and health care provider trust. In our sample, 63% of respondents reported a sense of relief at the idea of having an LARC. White and Hispanic/Latinx respondents were more likely than Black/African American respondents to report LARC relief. This finding is in line with previous research documenting racial/ethnic disparities in LARC attitudes [29–31]. Racial/ethnic differences were not attenuated by adjusting for differences in provider trust. So, while improving trust is important, it may not fully address racial/ethnic differences in LARC attitudes. Together, these patterns suggest that LARC perceptions are partly a joint function of individual-level fertility concerns and reversibility concerns that unfold in the wider social context of historically rooted racial/ethnic health care treatment inequalities that continues to shape patient attitudes today. Individual desires and preferences for contraception (not related to reversibility and fertility) likely explain some of these differences as well.

This study also has several limitations that provide opportunities for future research to address. As a cross-sectional study, which assessed patient attitudes before health care encounters, this study was unable to examine how factors associated with LARC relief may change across time or how patient attitudes may have changed following health care appointments. Additionally, the study’s small sample size made it likely underpowered to run race-stratified analysis or to analyze LARC relief among people of other racial/ethnic identities. Finally, as patients were recruited from a clinic-based study, this analysis may have limited generalizability to the population of low-income patients who may not have adequate access to health care even after the DelCAN intervention.

Despite these limitations, this study yields several important insights that may be useful to researchers and health care practitioners. Most respondents reported beliefs that IUDs and implants can cause future infertility (56% and 65%, respectively), and 27% also reported beliefs that IUDs are not reversible if the patient changes their mind. We found that a lack of LARC relief was associated with incorrect beliefs that IUDs and/or implants are not able to be reversed if the patient changes their mind and that IUDs may cause future infertility. The prevalence of incorrect beliefs regarding IUDs and implants among patients presents an opportunity for health care providers and public health practitioners to address through targeted health education interventions. Given our findings, designing interventions to educate patients on the reversibility and fertility effects of LARCs may promote efforts to equip people with the necessary information to make an informed choice about contraception that aligns with their preferences at that time.

The prevalence of the belief that IUDs are not reversible if the patient changes their mind is also likely due to practical concerns regarding a lack of access to reversibility as a form of contraceptive coercion [20]. Our findings underscore the significance of continued public health interventions to promote access to contraceptive health care among underserved patient populations. The strength of the associations between LARC relief and trust in health care providers to remove LARCs upon request also underscores the importance of the health care provider-patient relationship as the context within which contraceptive decisions are made.

## Supplementary Material

1

## Figures and Tables

**Table 1:** Characteristics of respondents in Title X sample (*N* = 797, Delaware, 2018–2019)

Variable	Mean or Percent

Race/ethnicity	
Black or African American	37%
White	31%
Hispanic/Latinx	31%
Age	26.73
Education	
Less than high school	20%
High school graduate	27%
Some college	31%
Associate’s degree	6%
Bachelor’s degree or more	16%
Insurance status	
Insured	74%
Uninsured	26%
Health care access and usage history	
Deferred health care in the past 12 mo	20%
No prior established health care in this setting	29%
Contraceptive attitudes	
LARC relief	63%
Belief that condoms are too much of a hassle	53%
Belief IUD cannot be removed early	27%
Belief that IUDs will impact future fertility	56%
Belief that implant will impact future fertility	65%
Provider trust	
Trust provider to remove IUD when requested	69%
Trust provider to remove implant when requested	70%
Wave 1	48%
Wave 2	52%

IUD = intrauterine device; LARC = long-acting reversible contraceptive. Characteristics reported pooling across cross-sectional surveys administered in 2018 and 2019. LARC relief refers to the variable indicating that respondents report a sense of relief at the idea of having a long-acting reversible contraceptive.

**Table 2:** Unadjusted proportions and correlations of LARC sense of relief across variables in Title X sample (*N* = 797, Delaware, 2018–2019)

Variable	Percent viewing LARC as relief	95% CI	

Race/ethnicity			
Black or African American	61%	55%	66%
White	66%	60%	72%
Hispanic/Latinx	64%	58%	70%
Education			
Less than high school	59%	52%	67%
High school graduate	59%	53%	66%
Some college	69%	63%	74%
Associate’s degree	66%	52%	79%
Bachelor’s degree or more	65%	56%	73%
Insurance status			
Insured	64%	60%	68%
Uninsured	61%	54%	68%
Health care access and usage history			
Did not defer health care in the past 12 mo	63%	59%	67%
Deferred health care in the past 12 mo	66%	58%	73%
No prior established health care in this setting	62%	56%	68%
Established health care in this setting	64%	60%	68%
Contraceptive attitudes			
Belief that condoms are too much of a hassle	63%	59%	68%
Belief that IUDs cannot be removed early	46%	39%	53%
Belief that IUDs will impact future fertility	56%	51%	61%
Belief that implant cannot be removed early	51%	44%	58%
Belief that implant will impact future fertility	59%	54%	63%
Provider trust			
Trust provider to remove IUD when requested	75%	71%	78%
Trust provider to remove implant when requested	74% Correlation with LARC relief	70%	78%
Age	−0.02		

IUD = intrauterine device; LARC = long-acting reversible contraceptive.

Characteristics reported pooling across cross-sectional surveys administered in 2018 and 2019. LARC relief refers to the variable indicating that respondents report a sense of relief at the idea of having a long-acting reversible contraceptive.

**Table 3:** Predicted probabilities estimating LARC sense of relief (*N* = 797, Delaware, 2018–2019)

	Unadjusted	Adjusted (Model 4)

Race/ethnicity		
Black/African American	60% (58%–63%)	59% (55%–63%)
White	66% (62%–71%)	67% (63%–71%)
Hispanic/Latinx	64% (60%–65%)	65% (62%–67%)
Did not trust provider to remove implant when requested	38% (34%–42%)	50% (43%–57%)
Trust provider to remove IUD when requested	75.0% (72.3%–77.4%)	70% (64%–76%)
Did not trust provider to remove implant when requested	38% (33%–43%)	52% (40%–63%)
Trust provider to remove implant when requested	74% (72%–76%)	69% (66%–72%)

IUD = intrauterine device.

Predicted probabilities reported as percentages calculated from unadjusted and fully adjusted logistic regression models using robust SEs to account for potential heteroskedasticity clustered by clinic location; CIs in parentheses. Adjusted probabilities are calculated from Model 4. When estimating the adjusted predicted probabilities, all other covariates are set at the sample mean.

## Appendix A. Supporting information

Supplementary data associated with this article can be found in the online version at doi:10.1016/j.contraception.2025.111002.
