# Supplementary material for: Disparities in perceptions of long-acting reversible contraceptives among patients in Delaware Title X clinics
Source: Contraception. Author manuscript; Available in PMC 2026 Jun 5. (PMC13240843; doi:10.1016/j.contraception.2025.111002)
Supplement: 1 [file NIHMS2162317-supplement-1.docx]

| Table 1: Characteristics of Respondents in Title X Sample (N=797, Delaware, 2018-2019) | |
| --- | --- |
|  | Mean or Percent |
| *Race/Ethnicity* | |
| Black or African American | 37% |
| White | 31% |
| Hispanic/Latinx | 31% |
| Age | 26.73 |
| *Education* | |
| Less than High School | 20% |
| High School graduate | 27% |
| Some college | 31% |
| Associate's degree | 6% |
| Bachelor's degree or more | 16% |
| *Insurance Status* |  |
| Insured | 74% |
| Uninsured | 26% |
| *Healthcare access and usage history* |  |
| Deferred healthcare in past 12 months | 20% |
| No prior established healthcare in this setting | 29% |
| *Contraceptive attitudes* |  |
| LARC relief | 63% |
| Belief that condoms are too much of a hassle | 53% |
| Belief IUD cannot be removed early | 27% |
| Belief that IUDs will impact future fertility | 56% |
| Belief that implant will impact future fertility | 65% |
| *Provider Trust* |  |
| Trust provider to remove IUD when requested | 69% |
| Trust provider to remove implant when requested | 70% |
| Wave 1 | 48% |
| Wave 2 | 52% |
| Notes: Characteristics reported pooling across cross-sectional surveys administered in 2018 and 2019. LARC relief refers to the variable indicating that respondents report a sense of relief at the idea of having a long-acting reversible contraceptive; IUD refers to intrauterine device. | |

| Table 2: Unadjusted Proportions and Correlations of LARC Sense of Relief across Variables in Title X Sample (N=797, Delaware, 2018-2019) | | | |
| --- | --- | --- | --- |
|  | Percent viewing LARC as Relief | 95% confidence interval | |
| *Race/Ethnicity* |  |  |  |
| Black or African American | 61% | 55% | 66% |
| White | 66% | 60% | 72% |
| Hispanic/Latinx | 64% | 58% | 70% |
| *Education* |  |  |  |
| Less than High School | 59% | 52% | 67% |
| High School graduate | 59% | 53% | 66% |
| Some college | 69% | 63% | 74% |
| Associate's degree | 66% | 52% | 79% |
| Bachelor's degree or more | 65% | 56% | 73% |
| *Insurance Status* |  |  |  |
| Insured | 64% | 60% | 68% |
| Uninsured | 61% | 54% | 68% |
| *Healthcare access and usage history* |  |  |  |
| Did not defer healthcare in past 12 months | 63% | 59% | 67% |
| Deferred healthcare in past 12 months | 66% | 58% | 73% |
| No prior established healthcare in this setting | 62% | 56% | 68% |
| Established healthcare in this setting | 64% | 60% | 68% |
| *Contraceptive attitudes* |  |  |  |
| Belief that condoms are too much of a hassle | 63% | 59% | 68% |
| Belief that IUDs cannot be removed early | 46% | 39% | 53% |
| Belief that IUDs will impact future fertility | 56% | 51% | 61% |
| Belief that implant cannot be removed early | 51% | 44% | 58% |
| Belief that implant will impact future fertility | 59% | 54% | 63% |
| *Provider Trust* |  |  |  |
| Trust provider to remove IUD when requested | 75% | 71% | 78% |
| Trust provider to remove implant when requested | 74% | 70% | 78% |
|  |  |  |  |
|  | Correlation with LARC Relief | |  |
| Age | -0.02 |  |  |

Notes: Characteristics reported pooling across cross-sectional surveys administered in 2018 and 2019. LARC relief refers to the variable indicating that respondents report a sense of relief at the idea of having a long-acting reversible contraceptive; IUD refers to intrauterine device.

Table 3: Predicted Probabilities Estimating LARC Sense of Relief (N=797, Delaware, 2018-2019)

|  | Unadjusted | Adjusted  (Model 4) |
| --- | --- | --- |
| Race/ethnicity | |  |
| Black/African American | 60% | 59% |
|  | (58%-63%) | (55%-63%) |
| White | 66% | 67% |
|  | (62%-71%) | (63%-71%) |
| Hispanic/Latinx | 64% | 65% |
|  | (60%-65%) | (62%-67%) |
|  |  |  |
| Did not trust provider to remove implant when requested | 38% | 50% |
|  | (34%-42%) | (43%-57%) |
| Trust provider to remove IUD when requested | 75.0% | 70% |
|  | (72.3%-77.4%) | (64%-76%) |
|  |  |  |
| Did not trust provider to remove implant when requested | 38% | 52% |
|  | (33%-43%) | (40%-63%) |
| Trust provider to remove implant when requested | 74% | 69% |
|  | (72%-76%) | (66%-72%) |

Notes: Predicted probabilities reported as percentages calculated from unadjusted and fully adjusted logistic regression models using robust standard errors to account for potential heteroskedasticity clustered by clinic location; confidence intervals in parentheses. Adjusted probabilities are calculated from Model 4. When estimating the adjusted predicted probabilities, all other covariates are set at the sample mean.

Table S1: Missing and Imputed Data for Each Variable

|  | Nonmissing observations | Imputed percent |
| --- | --- | --- |
| Race/ethnicity | 720 | 9.7% |
| Insurance status | 768 | 3.6% |
| Education | 789 | 1.0% |
| Age | 713 | 10.5% |
| Wave | 797 | 0.0% |
| Deferred | 720 | 9.7% |
| Established care | 793 | 0.5% |
| condoms too much of a hassle | 794 | 0.4% |
| IUD reversibility concerns | 676 | 15.2% |
| IUD fertility concerns | 708 | 11.2% |
| Implant fertility concerns | 659 | 17.3% |
| Trust provider to remove IUD when requested | 709 | 11.0% |
| Trust provider to remove implant when requested | 657 | 17.6% |

Table S2: Factors associated with LARC sense of relief. (Complete Case Analysis, N=471, Delaware, 2018-2019)

|  | Model 1 | Model 2 | Model 3 | Model 4 |
| --- | --- | --- | --- | --- |
| *Race/Ethnicity (ref. Black/African American)*  White | 1.27* | 1.39* | 1.26 | 1.38+ |
|  | (1.02 - 1.59) | (1.06 - 1.80) | (0.82 - 1.94) | (0.99 - 1.92) |
| Hispanic/Latinx | 1.66*** | 1.90*** | 1.76** | 1.70** |
|  | (1.36 - 2.03) | (1.41 - 2.57) | (1.22 - 2.52) | (1.20 - 2.42) |
| Age | 1.00 | 0.99 | 0.99 | 0.99 |
|  | (0.98 - 1.02) | (0.98 - 1.01) | (0.97 - 1.01) | (0.97 - 1.02) |
| Survey wave (ref. wave 1) | 0.70+ | 0.69* | 0.70 | 0.68 |
|  | (0.49 - 1.01) | (0.48 - 0.98) | (0.37 - 1.35) | (0.34 - 1.37) |
| *Education (ref. less than High School)*  High School graduate |  | 0.66 | 0.60+ | 0.66 |
|  |  | (0.37 - 1.17) | (0.35 - 1.06) | (0.36 - 1.22) |
| Some college |  | 1.08 | 1.05 | 1.39 |
|  |  | (0.67 - 1.75) | (0.61 - 1.81) | (0.86 - 2.25) |
| Associate's degree |  | 1.37 | 1.17 | 1.31 |
|  |  | (0.40 - 4.69) | (0.34 - 4.10) | (0.35 - 4.84) |
| Bachelor's degree or more |  | 0.89 | 0.83 | 1.11 |
|  |  | (0.50 - 1.59) | (0.43 - 1.62) | (0.59 - 2.07) |
| Uninsured (ref. insured) |  | 0.70** | 0.75 | 0.82 |
|  |  | (0.55 - 0.90) | (0.51 - 1.11) | (0.47 - 1.44) |
| *Healthcare access and usage history*  Deferred care |  | 1.26 | 1.27 | 1.47 |
|  |  | (0.80 - 1.98) | (0.92 - 1.74) | (0.89 - 2.41) |
| Established care |  | 1.21 | 1.18 | 1.20 |
|  |  | (0.91 - 1.61) | (0.83 - 1.67) | (0.78 - 1.85) |
| *Contraceptive attitudes*  Belief that condoms are too much of a hassle |  |  | 1.07 | 1.19+ |
|  |  |  | (0.94 - 1.22) | (0.99 - 1.42) |
| Belief that IUDs cannot be removed early |  |  | 0.35*** | 0.62** |
|  |  |  | (0.25 - 0.48) | (0.44 - 0.88) |
| Belief IUD causes infertility |  |  | 0.56** | 0.63** |
|  |  |  | (0.39 - 0.80) | (0.46 - 0.86) |
| Belief that implant will impact future fertility |  |  | 0.93 | 1.06 |
|  |  |  | (0.41 - 2.11) | (0.42 - 2.67) |
| *Provider trust*  Trust provider to remove IUD when requested |  |  |  | 2.80*** |
|  |  |  |  | (1.74 - 4.52) |
| Trust provider to remove implant when requested |  |  |  | 2.40*** |
|  |  |  |  | (1.59 - 3.60) |

Notes: Odds ratios reported; 95% confidence intervals in parentheses; +p<0.10, *p<0.05, **p<0.01, ***p<0.001

Table S3: Factors associated with LARC sense of relief. (N=797, Delaware, 2018-2019)

|  | Model 1 | Model 2 | Model 3 | Model 4 |
| --- | --- | --- | --- | --- |
| *Race/Ethnicity (ref. Black/African American)*  White | 1.29* | 1.36** | 1.30+ | 1.49** |
|  | (1.05 - 1.59) | (1.09 - 1.71) | (0.99 - 1.70) | (1.13 - 1.97) |
| Hispanic/Latinx | 1.21* | 1.45*** | 1.36** | 1.34* |
|  | (1.04 - 1.41) | (1.21 - 1.73) | (1.12 - 1.66) | (1.05 - 1.72) |
| Age | 0.99 | 0.99 | 0.99 | 0.99 |
|  | (0.98 - 1.01) | (0.97 - 1.01) | (0.97 - 1.01) | (0.97 - 1.01) |
| Survey wave (ref. wave 1) | 0.88 | 0.87 | 0.93 | 0.95 |
|  | (0.71 - 1.10) | (0.73 - 1.03) | (0.83 - 1.04) | (0.87 - 1.04) |
| *Education (ref. less than High School)*  High School graduate |  | 1.04 | 0.95 | 1.01 |
|  |  | (0.72 - 1.49) | (0.59 - 1.53) | (0.57 - 1.79) |
| Some college |  | 1.61** | 1.49* | 1.84** |
|  |  | (1.13 - 2.28) | (1.01 - 2.19) | (1.24 - 2.71) |
| Associate's degree |  | 1.75* | 1.57+ | 1.80** |
|  |  | (1.03 - 2.97) | (0.96 - 2.57) | (1.19 - 2.73) |
| Bachelor's degree or more |  | 1.30+ | 1.07 | 1.33 |
|  |  | (0.99 - 1.69) | (0.79 - 1.44) | (0.92 - 1.92) |
| Uninsured (ref. insured) |  | 0.79*** | 0.81* | 0.88 |
|  |  | (0.69 - 0.90) | (0.68 - 0.97) | (0.67 - 1.14) |
| *Healthcare access and usage history*  Deferred care |  | 1.14 | 1.18 | 1.28 |
|  |  | (0.74 - 1.76) | (0.81 - 1.73) | (0.84 - 1.93) |
| Established care |  | 1.16 | 1.11 | 1.11 |
|  |  | (0.92 - 1.47) | (0.87 - 1.41) | (0.85 - 1.43) |
| *Contraceptive attitudes*  Belief that condoms are too much of a hassle |  |  | 1.08 | 1.18 |
|  |  |  | (0.85 - 1.37) | (0.91 - 1.54) |
| Belief that IUDs cannot be removed early |  |  | 0.45*** | 0.77+ |
|  |  |  | (0.35 - 0.58) | (0.58 - 1.03) |
| Belief IUD causes infertility |  |  | 0.69** | 0.82 |
|  |  |  | (0.54 - 0.88) | (0.62 - 1.08) |
| Belief that implant will impact future fertility |  |  | 0.73 | 0.83 |
|  |  |  | (0.46 - 1.15) | (0.52 - 1.32) |
| *Provider trust*  Trust provider to remove IUD when requested |  |  |  | 2.53*** |
|  |  |  |  | (1.47 - 4.35) |
| Trust provider to remove implant when requested |  |  |  | 2.25** |
|  |  |  |  | (1.28 - 3.94) |

Notes: Odds ratios reported; 95% confidence intervals in parentheses; +p<0.10, *p<0.05, **p<0.01, ***p<0.001
